# Pleural Tuberculosis: A Febrile Presentation Without Respiratory Symptoms

**DOI:** 10.7759/cureus.10643

**Published:** 2020-09-25

**Authors:** Ussama Ghumman, Haider Ghumman, Khalid Nawab, Amandeep Singh, Awais Naeem

**Affiliations:** 1 Internal Medicine, Geisinger Commonwealth School of Medicine, Camp Hill, USA; 2 Internal Medicine, University of South Florida Morsani College of Medicine, Tampa, USA; 3 Internal Medicine, Geisinger Holy Spirit Hospital, Camp Hill, USA; 4 Internal Medicine, Khyber Medical University, Peshawar, PAK

**Keywords:** tuberculosis, pleural tuberculosis, pleural effusion, fever

## Abstract

Tuberculosis (TB) is one of the largest public health crises globally, with pleural TB comprising a large portion of cases. It has a significantly minimal presence within the United States in comparison to the rest of the world. Awareness of its presence and acumen on diagnostics and treatment are essential. Conventional tests are often time consuming, and do not always yield accurate results. We present the case of a patient presenting with fevers but no cough, who eventually found to have large pleural effusion and concluded to have pleural TB without pulmonary parenchymal involvement. He then showed measurable improvement with empiric treatment.

## Introduction

Mycobacterium tuberculosis (MTB) is an extensively studied bacterium. Tuberculosis (TB) remains the leading infectious cause of death worldwide, and pleural tuberculosis (PT) remains one of the most frequent causes of pleural exudates [1]. The primary mode of transmission is through aerosol droplets, which enter the alveolar passages of exposed individuals. The TB bacteria then takes residence in a variety of cells, including resident macrophages, alveolar epithelial type II pneumocytes, and dendritic cells, which mediate its spread throughout the body [2]. Presentations of the disease are variable, ranging from classic caseous presentations to pleural exudative ones. Lazarus et al. reported that TB is a front runner in causing pleural effusions globally comprising 30%-60% of all pleural effusions; however, in the United States, the number is only 2%-5% that is comparably minuscule [3]. Epidemiological studies on TB have shown a significant male predominance and increased predilection for the 20- to 39-year-old age group, and in those living within cities [4]. Diabetes mellitus, a positive family history, and prior infection are the greatest risk factors for TB, with common symptoms including recurrent fever, cough, anorexia, and weight loss [5]. Treatment can be challenging, as MTB “exhibits a clonal population structure with low DNA sequence diversity”, yielding drug resistance and difficulties in pinning down genome sequences [6]. Understanding the pathogenesis, epidemiology, approach to diagnosis, and treatment of PT are necessary in tackling this illness.

## Case presentation

A 30-year-old male with no past medical history, who does not smoke, drink, or use any other substance of abuse, presented to the emergency room (ER) with two weeks of unremitting waxing and waning fever. He had presented to an outpatient urgent care facility two weeks prior where he was told the etiology of his fever was viral and sent home with ibuprofen and acetaminophen. The fever persisted despite round-the-clock usage of antipyretics. He was ill appearing with associated symptoms, including poor appetite, reported five-pound weight loss, night sweats, and an episode of non-bloody vomiting. He had no associated upper respiratory symptoms, including cough, rhinorrhea, congestion, or sore throat, and denied hemoptysis. The patient emigrated from Pakistan and had been living in the United States for two years with no recent travel out of the country.

Upon initial physical examination, breath sounds were decreased at the left middle and lower lung fields, without wheezing, rhonchi or rales. Initial blood workup was unrevealing, with normal levels of white blood cells (6.5 K/µL), hemoglobin, hematocrit, and platelets. Renal function, electrolytes, and liver function were intact. Urinalysis showed no abnormalities, respiratory viral panel was negative, and electrocardiogram showed no acute abnormal findings.

**Figure 1:**
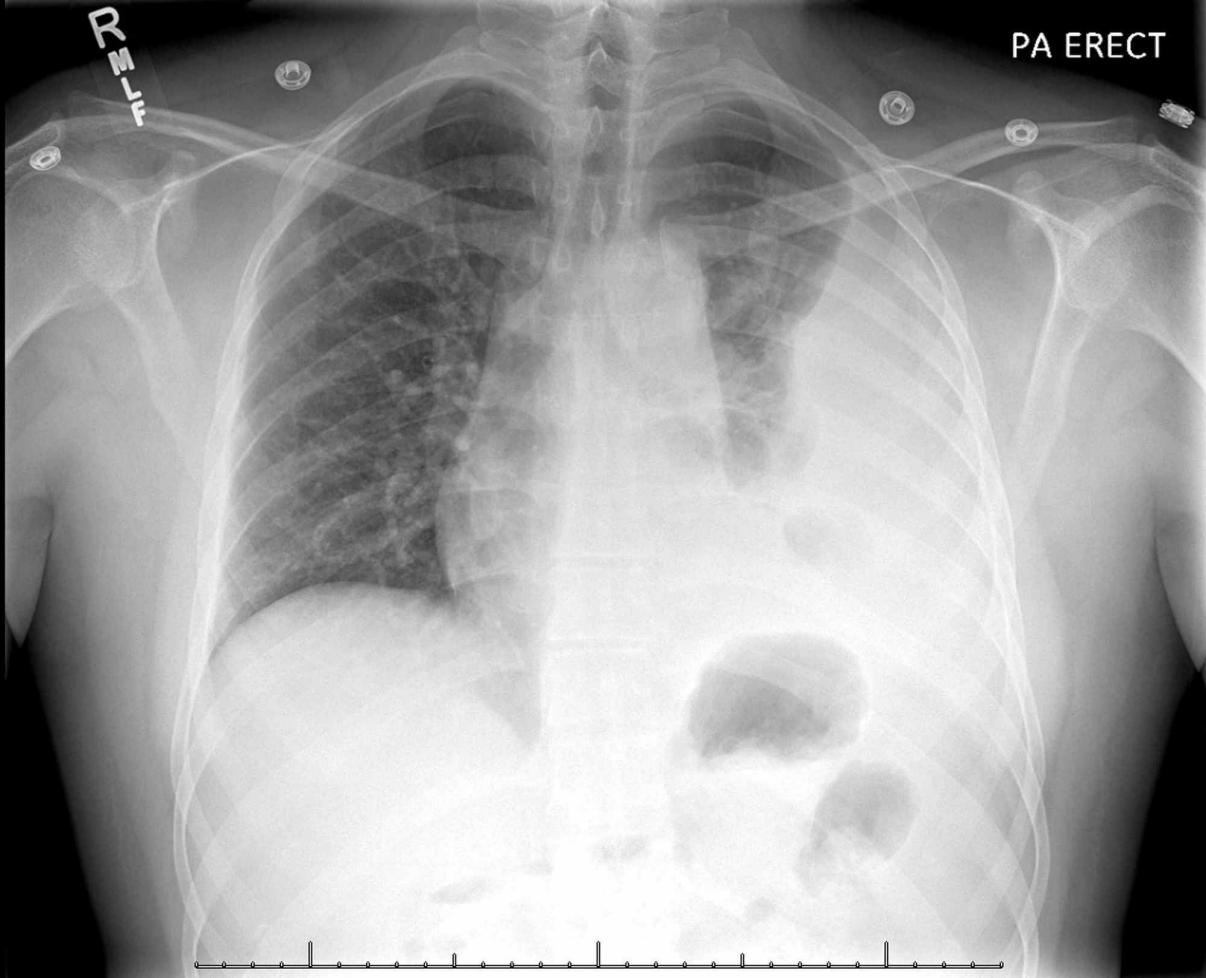
Initial chest x-ray of the patient showing left-sided pleural effusion with atelectasis.

**Figure 2:**
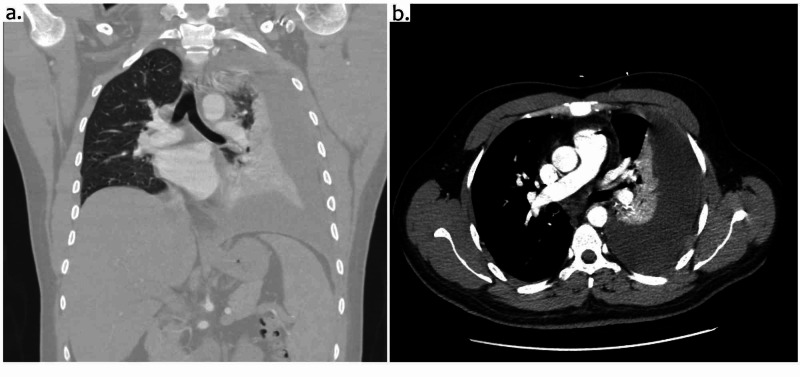
CT scan of the chest: coronal plane (a) and cross-sectional plane (b) showing large pleural effusion on the left side with atelectasis of the left lung.

X-ray of the chest showed large loculated pleural effusion with atelectasis/consolidation of the left lung concerning for possible pneumonia (Figure 1). CT scan of the chest revealed a large non-hemorrhagic left pleural effusion, with lower and upper lobe atelectasis (Figure 2). No pneumothorax, hematoma, mass, or adenopathy was seen, and the right lung elicited no findings. The patient was admitted under the impression of pneumonia with no associated cough or dyspnea, and was started on intravenous (IV) azithromycin and ceftriaxone, ipratropium bromide and albuterol sulfate nebulizer, antipyretics (ibuprofen and acetaminophen), and IV fluids.

The patient relayed that he had testing for latent TB done before coming to the United States which was negative. Upon infectious disease (ID) workup, suspicion for pulmonary TB was low. Legionella antigen, human immunodeficiency virus (HIV) screening, viral hepatitis panel, and urinary strep antigen were negative. The patient underwent left-sided thoracentesis for possible empyema, draining 1,300 milliliters of clear yellow fluid. The fluid analysis was consistent with that of an exudate. Gram stain was negative for any organism. The fluid was lymphocytic predominant with 77% lymphocytes, with high lactate dehydrogenase (LDH) and low glucose, raising concerns for TB.

His fever continued and at this point, the patient was put on airborne precautions. IV vancomycin was added to cover empirically for methicillin-resistant Staphylococcus aureus (MRSA) pneumonia. Repeat chest x-ray showed improvement in left-sided pleural effusion with a persistent left lower lobe opacity (likely a combination of residual effusion and atelectasis). The pulmonary specialist recommended a pleural biopsy as bacterial stains had been negative. Further testing showed positive QuantiFERON-TB® Gold testing, negative autoimmune workup, negative pleural fluid cytology, and elevated pleural fluid adenosine deaminase (ADA) level (55.8 U/L). Keeping this workup as well as the fact that he recently migrated from Pakistan and had lymphocytic predominant exudative pleural effusion, it was decided to start him on anti-TB therapy empirically. Rifampin 600 mg daily, isoniazid 300 mg daily, pyrazinamide 2,000 mg daily, ethambutol 1,600 mg daily (RIPE), and vitamin B6 50 mg daily were started for presumptive PT. Vancomycin, ceftriaxone, and azithromycin were stopped. A pleural biopsy was considered as an elevated ADA can be present with lymphoma, mesothelioma, metastatic malignancy, and TB. However, cardiothoracic surgery recommended transfer to a higher level of care hospital where bronchoscopy and pleural biopsy can be done at the same time. The patient was transferred to another hospital for further management. Repeat thoracentesis at the second facility showed negative acid-fast bacillus (AFB) on smear but elevated ADA. Considering his fevers had resolved with RIPE therapy and pleural effusion seemed to be subsiding, it was decided to do only bronchoscopy with bronchoalveolar lavage (BAL) to rule out pulmonary TB and not do the pleural biopsy. He underwent bronchoscopy with BAL, which came back negative for AFB. It was concluded that patient had isolated PT with no pulmonary involvement, not requiring any isolation. He was then discharged home with continuation of RIPE therapy.

**Figure 3:**
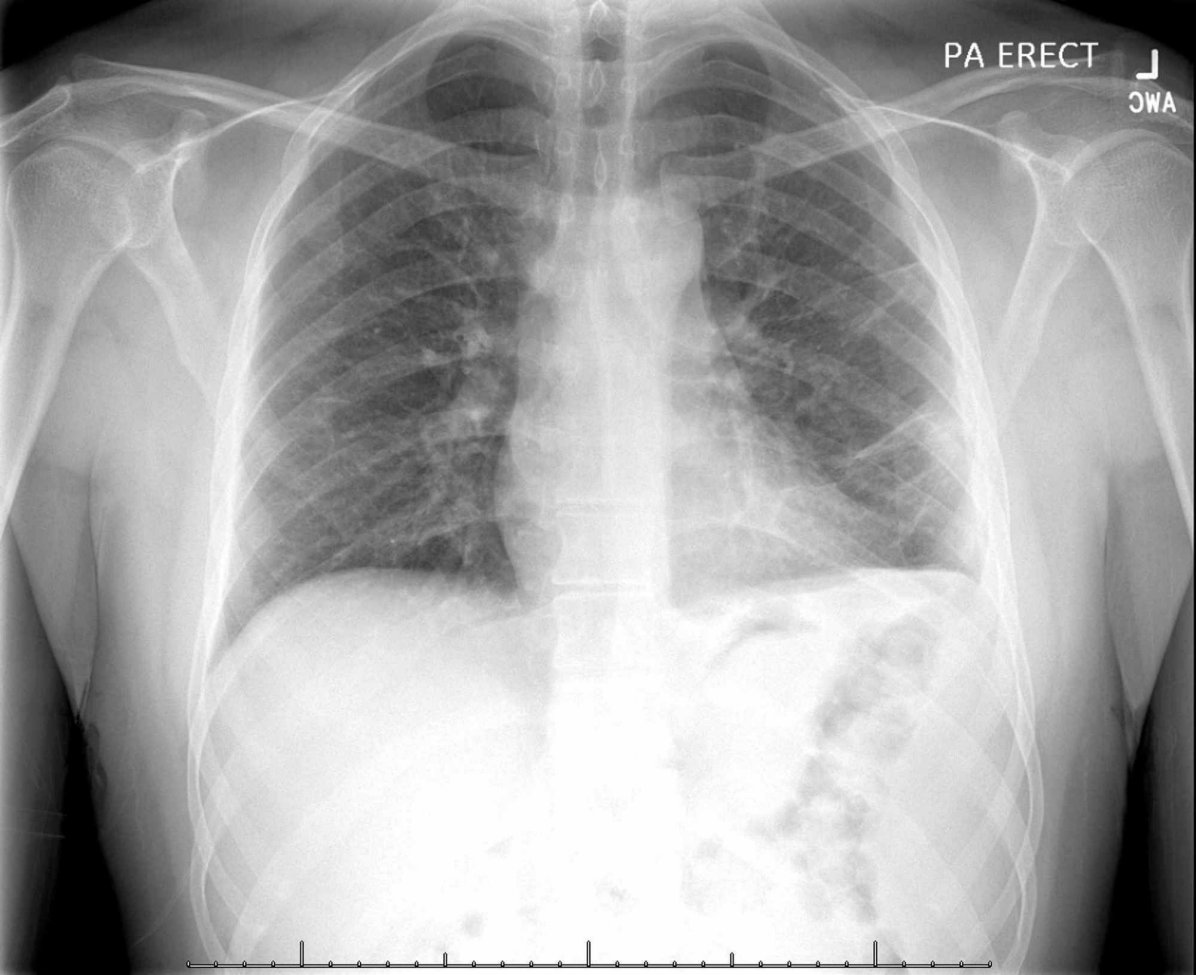
Follow-up chest x-ray, months into anti-tuberculous therapy showing significant improvement.

On three-month follow-up, the patient reported mild intermittent left lower chest pain, but remained afebrile with no other symptoms. Blood work was grossly unremarkable at this time with exception of mildly increased liver enzymes, which were determined to be secondary to anti-TB medication. His chest x-ray showed significant improvement compared to his initial presentation (Figure 3).

## Discussion

TB has high morbidity and mortality worldwide; thus, it is imperative to understand the geographic presence and the diagnostic arsenal available [7]. It makes up less than 1% of exudative effusions in the west but the numbers are staggering in nations such as India and Pakistan, as well as other developing nations. It is hypothesized that TB can be the culprit of up to 80% of pleural effusions in these locations [8]. The epidemiology and traditional demographics of TB haVe been shifting with more data showing increased presence with HIV co-infection and those that are immunosuppressed [9]. PT is the most common extrapulmonary form in adults [10].

Common clinical presentations of PT may include cough, chest pain, fever, malaise, fatigue, anorexia, weight loss, night sweats, and dyspnea. Chest x-ray may show findings such as cavitation’s (in case of parenchymal involvement) and consolidations that are the hallmarks of active disease. Tuberculin skin tests are useful in low-prevalence areas. A positive test has high diagnostic value, but tuberculin skin tests may be negative in 30%-40% of patients with the disease process [10]. Some of the tools at our disposable such as microbiological tests have been shown to have low sensitivity. Tyagi et al. find that microbiological tests “are usually found to be inadequate for pleural tuberculosis diagnosis” [11]. AFB are rarely found in sputum, and often multiple sputum smears may be negative, or a patient may not be able to expectorate appropriately if there is no parenchymal involvement. This often leads to the next step being BAL and bronchoscopy [12]. One study elicited a sensitivity of 57.1% for BAL smear, and a sensitivity of 76.7% for post-bronchoscopy smear [13]. These findings have shown that bronchoscopy and BAL are both viable and available avenues of diagnosis.

Pleural fluid analysis has been an established part of diagnosing PT. In the presented case, a large number of lymphocytes within the pleural fluid are often considered diagnostic [14]. However, a small portion of PT has also shown to be neutrophil predominant. A recent retrospective analysis, however, showed stronger inflammatory reactions from neutrophilic pleurisy [15]. This calls into question the traditional algorithm and emphasizes the importance of a thorough workup. In concomitance with lymphocyte-rich pleural fluid, high ADA is a common and highly sensitive finding within endemic areas. Levels higher than 40 U/L are often diagnostic [16]. Measuring levels of interferon-gamma have also yielded useful diagnostic results [17]. Negative ADA and lack of lymphocytic predominance make TB very unlikely, with the next step being pleural biopsy [1].

Even without a definitive diagnosis, anti-tuberculous therapy is imperative in patients with appropriate epidemiology supported by diagnostic studies or as a diagnosis of exclusion. Pleural drainage via thoracentesis, as done in this patient twice, is an appropriate adjunct to medical management [18]. Treatment for PT is similar to that of pulmonary parenchymal TB, with two months of induction therapy followed by maintenance therapy depending on local resistance pattern [19].

## Conclusions

PT is a well-known entity and may present without involvement of pulmonary parenchyma, obscuring the diagnosis. Therefore, if the patient has epidemiological risk factors, they should be worked up for PT. Pleural fluid AFB smears and cultures may have poor yield; however, high pleural fluid ADA (>40 U/L) should raise concerns for PT and in the presence of epidemiological risk factors, anti-TB treatment should be considered. 
